# LIGHTS! CAMERA! REPRESENTATION!: EXAMINING AN INTERGENERATIONAL FILM COURSE FOCUSED ON MEDIA REPRESENTATION

**DOI:** 10.1093/geroni/igad104.2069

**Published:** 2023-12-21

**Authors:** Samuel Van Vleet

**Affiliations:** Miami University, Oxford, Ohio, United States

## Abstract

This paper explores the humanities-based approach to an intergenerational course focused on providing older adults with a guided course in filmmaking. The goals for this course were to provide opportunities for intergenerational lifelong learning, insight into older adult led media representation, and intergenerational community engagement. This course was hosted through an Institute for Learning in Retirement. Throughout the course, students were taught the fundamentals of filmmaking and storytelling. The class worked together to create an original short film screenplay that focused on topics related to their perspectives as older adults. After the script was completed, older adult students were introduced to undergraduate students from a local university that assisted in the creation of their short film. The film equipment and resources were provided to the students which allowed the students to focus on working together to create an original short film. After the film was completed, it was premiered to the community which included a post-film Q&A session that allowed the students to describe their experience working on this intergenerational project. The Q&A session also focused on how important it was to the older adult students to have a role in creating media representation of an older population. This paper highlights the impact of providing opportunities for older adult representation through a creative process while also promoting opportunities for intergenerational learning related to narrative work.

